# CCNF is a potential pancancer biomarker and immunotherapy target

**DOI:** 10.3389/fonc.2023.1109378

**Published:** 2023-04-24

**Authors:** Lifang Wei, Huiming Li, Mengjun Xiao, Cuiling Zhou, Jiliang Liu, Shilian Weng, Ruda Wei

**Affiliations:** ^1^ Shenzhen Traditional Chinese Medicine Hospital, The Fourth Clinical Medical College of Guangzhou University of Chinese Medicine, Shenzhen, China; ^2^ Department of Preventive Medicine, Medical School of Yichun University, Yichun, China; ^3^ Oncology Department, Shenzhen Overseas Chinese Hospital Affiliated to Jinan University, Shenzhen, China

**Keywords:** CCNF, pancancer, bioinformatics, immune cell infiltration, methylation, biomarker

## Abstract

**Background:**

CCNF catalyzes the transfer of ubiquitin molecules from E2 ubiquitin-conjugating enzymes to target proteins, thereby regulating the G1/S or G2/M transition of tumor cells. Thus far, CCNF expression and its potential as a pancancer biomarker and immunotherapy target have not been reported.

**Methods:**

TCGA datasets and the R language were used to analyze the pancancer gene expression, protein expression, and methylation levels of CCNF; the relationship of CCNF expression with overall survival (OS), recurrence-free survival (RFS), immune matrix scores, sex and race; and the mechanisms for posttranscriptional regulation of CCNF.

**Results:**

CCNF expression analysis showed that CCNF mRNA expression was higher in cancer tissues than in normal tissues in the BRCA, CHOL, COAD, ESCA, HNSC, LUAD, LUSC, READ, STAD, and UCEC; CCNF protein expression was also high in many cancer tissues, indicating that it could be an important predictive factor for OS and RFS. CCNF overexpression may be caused by CCNF hypomethylation. CCNF expression was also found to be significantly different between patients grouped based on sex and race. Overexpression of CCNF reduces immune and stromal cell infiltration in many cancers. Posttranscriptional regulation analysis showed that miR-98-5p negatively regulates the expression of the CCNF gene.

**Conclusion:**

CCNF is overexpressed across cancers and is an adverse prognostic factor in terms of OS and RFS in many cancers; this phenomenon may be related to hypomethylation of the CCNF gene, which could lead to cancer progression and worsen prognosis. In addition, CCNF expression patterns were significantly different among patients grouped by sex and race. Its overexpression reduces immune and stromal cell infiltration. miR-98-5p negatively regulates CCNF gene expression. Hence, CCNF is a potential pancancer biomarker and immunotherapy target.

## Introduction

Globally, there were approximately 19.3 million new cancer cases and 10 million related deaths worldwide in 2020, and morbidity and mortality rates are rising rapidly year by year (1). Cancer presents the greatest social and economic burden among all human diseases, and lung cancer, breast cancer and prostate cancer are the top three (2). In recent years, the discovery of new tumor markers and immunotherapeutic targets has become increasingly important in the early diagnosis and treatment of cancer (3, 4). Precision medicine based on tumor markers and immune targets provides a new strategy for cancer research (5).

Cyclin F (CCNF, also known as FBXO1), a founding member of the F-box protein family, was first reported in 1994. It is located in the nucleus and participates in the transfer of ubiquitin molecules from the E2 ubiquitin binding enzyme to the target protein catalyzed by the E3 ubiquitin ligase Skp1-Cul1-F-box (SCF) through the F-box motif (6). CCNF expression oscillates during the cell cycle and reaches its peak only in the G2 phase (7). Studies have shown that CCNF not only regulates the transformation of tumor cells from G1 to S phase by affecting the expression of E2F family genes (8) but also promotes the degradation of E2F7/8 protein *via* ubiquitination to induce the transition of tumor cells from the G2 phase to the M phase, and both of these processes occur independent of the interaction between cyclin-dependent kinase (CDK) and anaphase-promoting complex/cyclosome (APC/C) (9). Moreover, CCNF regulates the cell cycle and maintains the stability of the genome and dNTP pool by promoting the degradation of ribonucleotide reductase subunit M2 (RRM2) (10, 11). According to DESHMUKH R S, CCNF reduced the carcinogenicity of the R132H isocitrate dehydrogenase 1 (IDH1) mutation by directly downregulating the recombination signal binding protein for immunoglobulin kappa J region (RBPJ) (12). The report from FU J indicated that the low expression of CCNF led to poor differentiation and a poor prognosis in hepatocellular carcinoma (13). These two reports suggest that CCNF is likely to be a tumor suppressor gene. However, studies have also shown that the overexpression of CCNF predicts a poor prognosis in ovarian cancer (14), liver cancer (15) and skin melanoma (16), suggesting that CCNF is an oncogene. Although contradictory, the above reports revealed that the functions of CCNF are likely to be related to the state of cells (11). In addition, the expression level of CCNF is closely related to the activity of cells in human tissues, mainly in the lung, skin and immune system (17). Therefore, CCNF is a potential biomarker and immunotherapy target for multiple cancers.

With the development of genetics and cancer genomics technologies, it has been recognized that key gene mutations, mutations in signaling cascades and immunological changes have certain genetic commonness and specificity in different types of cancer. Pancancer analysis not only has very important guiding significance for the diagnosis and treatment of different types of cancer (18) but also greatly facilitates the study of posttranscriptional regulation in cancer (19). The Cancer Genome Atlas (TCGA) pancancer dataset provides publicly available human gene expression profiles for 33 cancer types (20), which lays a foundation for cross-cancer studies of the molecular and pathological features of tumors and the corresponding clinical characteristics of patients (21). Recently, through pancancer analysis based on the TCGA database, molecular changes between cancer and normal tissues have been discovered at the genome, transcriptome and proteome levels in many cancers.

At present, there are only a few single-cancer analyses on the correlation between CCNF and cancer features using the TCGA database. Although the pancancer expression of CCNF and its effect on clinical prognosis have not been reported, existing studies suggest that there may be a close relationship between CCNF and the occurrence and development of cancer.

In this study, bioinformatics analysis based on the TCGA database was performed to analyze the correlation between CCNF expression level and survival, sex, race, clinicopathological stage, tumor mutation burden (TMB), microsatellite instability (MSI) status, DNA methylation level, single nucleotide variation (SNV), copy number variation (CNV), immune cell infiltration, immune matrix score and immune checkpoint gene expression across cancers. The CCNF protein-protein interaction (PPI) network was generated, and Gene Ontology (GO) and Kyoto Encyclopedia of Genes and Genomes (KEGG) enrichment analysis was performed. The mechanism of CCNF posttranscriptional regulation and the basic physical and chemical properties and spatial structure of CCNF were also analyzed. The results of this study could provide helpful information for further exploring the complex relationship between CCNF and multiple cancer types.

## Materials and methods

### Comparison of CCNF gene expression and protein expression levels in pancancer tissues, normal tissues or adjacent tissues

The expression level of CCNF in 33 kinds of cancer tissues and adjacent normal tissues from TCGA database was statistically analysed by Wilcoxon test *via* TIMER 2.0 (22) (http://timer.cistrome.org/). Using GEPIA2 (23) (http://gepia2.cancer-pku.cn/#index) based on the standardized calculation method of the UCSC Xena database (24) (http://xena.ucsc.edu/), the RNA-seq expression of CCNF in 33 kinds of cancer tissues in the TCGA database and normal tissues in the GTEx database was analysed by one-way ANOVA. The RNA expression of CCNF in 33 kinds of cancer tissues and normal tissues or paracancerous tissues was analysed by Mann-Whitney U test by Xiantao academic (https://www.xiantao.love/) based on R language 3.6.3 ggplot2 package and RNA-seq data. In addition, The Human Protein Atlas (HPA) database (25) (https://www.proteinatlas.org/) was used to assess CCNF protein expression in the pancancer tissues and normal tissues; the HPA071600 antibody-stained cancer tissue and normal tissue specimens were selected for immunohistochemistry analysis.

### Effect of CCNF expression on patient survival in multiple cancer types

Through Kaplan-Meier Plotter (26) (https://kmplot.com/analysis/) based on the GEO, EGA and TCGA databases, the correlation of CCNF mRNA expression with OS and RFS in 21 cancer types was evaluated. The gene chip data of 33 pancancer types were downloaded through UCSC Xena, and the R language 4.2.1 ggforest package was used to perform Cox regression analysis of factors affecting survival. PrognoScan (27) (http://dna00.bio.kyutech.ac.jp/PrognoScan/index), which contains cancer microarray data, was first applied to group patients into high and low CCNF expression groups *via the* minimum p value method and was then used to evaluate the correlation between CCNF expression and the survival of the two groups by log-rank test.

### Effect of sex and race on the expression of CCNF across cancers

The expression of CCNF in different sex and race subgroups in the pancancer cohort was compared using UALCAN (28) (http://ualcan.path.uab.edu/); the expression of CCNF estimated by the RSEM algorithm based on the 31 cancer types transcripts RNA-seq data downloaded from R 3.2.2 TCGA-Assembler, and the clinical data regarding sex and race were obtained from Genomic Data Commons (GDC) (https://gdc.cancer.gov/) to analyse the effect of sex and race on pancancer CCNF expression.

### Correlation between expression of CCNF and clinicopathological stage

The correlation between mRNA expression of CCNF and clinicopathological stage across cancers was analysed *via* GSCA (29) (http://bioinfo.life.hust.edu.cn/GSCA/#/); pathological, clinical, Masaoka and IGCCCG stage data for 27 cancer types were assessed *via* Wilcoxon test (number of subgroups=2) or ANOVA test (number of subgroups>2) and Mann-Kendall Trend Test. The expression of CCNF in pancancer and clinical stages were analysed by one-way ANOVA by GEPIA2.

### Correlation between CCNF expression and TMB and MSI in pancancer

The correlation of CCNF expression with TMB and MSI across cancers was analyzed by the R language 4.2.1 ggpubr package based on the gene chip data of 33 cancer types downloaded from the UCSC Xena database.

### Assessment of the methylation level of CCNF in pancancer tissues and its effect on survival/prognosis and prediction of the microRNA that post transcriptionally regulates CCNF

The methylation level of CCNF in pancancer tissues was compared with that in normal tissues by UALCAN. MethSurv (30) (https://biit.cs.ut.ee/methsurv/) was used for multivariate analysis of factors affecting survival based on DNA methylation data for 7358 patients from the TCGA GDAC Firehose dataset (with 25 types of cancer) and to analyze the effect of CCNF methylation level on survival/prognosis of pancancer patients. TargetScanHuman 8.0 (31) (http://www.targetscan.org/vert80/) was used to predict the microRNAs that target CCNF based on the presence of conserved loci in the 3’-untranslated region (3’-UTR) in *Homo sapiens*.

### SNV and CNV of the CCNF gene across cancers

The SNV and CNV of the CCNF gene in pancancer were analyzed by GSCA.

### Correlation between the expression of CCNF and immune cell infiltration, immune score, matrix score, and immune checkpoint gene expression across cancers

With R version 4.2.1 and the CIBERSORT algorithm, gene chip data for 33 cancer types downloaded from the UCSC Xena database were analyzed to determine the correlation between the expression of CCNF and the infiltration of 22 kinds of immune cells across cancers. The relationship between the expression of CCNF and the immune score and matrix score was analyzed *via the* ESTIMATE algorithm in R version 4.2.1. The expression of the CCNF gene and immune checkpoint genes was extracted by the R language 4.2.1 limma package. The correlation between the CCNF gene and immune checkpoint genes was calculated by the cor.test function. Then, the correlations between the CCNF gene and immune checkpoint genes were mapped *via the* ggpubr, ggExtra and ggplot2 packages and visualized *via the* ComplexHeatmap package.

### PPI network and GO and KEGG enrichment analyses of CCNF

STRING 11.5 (32) (https://cn.string-db.org/) was used to predict the PPI network of CCNF, setting the species as “Homo sapiens” and the minimum combined score as 0.4. The top 5 hub coding genes in the PPI network were screened by the cytoHubba app of Cytoscape software (33). The top 5 hub genes were further enriched and analyzed by GO and KEGG through the R version 4.2.1 packages limma, org.Hs.eg.db, cluster Profiler, and enrichplot.

### Prediction of basic physical and chemical properties and secondary structure of the CCNF protein

The amino acid sequence of the CCNF protein in *Homo sapiens* was obtained from the NCBI database (https://www.ncbi.nlm.nih.gov/). The physical and chemical properties of the CCNF protein were predicted by ProtParam (https://web.expasy.org/protparam/). The hydrophilicity of the CCNF protein was predicted by ProtScale (https://web.expasy.org/protscale/). The transmembrane topology of the CCNF protein was predicted by TMHMM 1.0.12 (https://dtu.biolib.com/DeepTMHMM). The phosphorylation sites of serine, threonine and tyrosine residues in the CCNF protein were predicted by NetPhos 3.1 (34) (https://services.healthtech.dtu.dk/service.php?NetPhos-3.1). The signal peptide of the CCNF protein was predicted by SignalP 6.0 (35) (https://services.healthtech.dtu.dk/service.php?SignalP). The secondary structure of the CCNF protein was predicted by SOPMA (https://npsa-prabi.ibcp.fr/cgi-bin/npsa_automat.pl?page=npsa_sopma.html).

### Prediction of the tertiary structure, enzyme binding activity, and ligand binding sites of the CCNF protein

The tertiary structure data of the CCNF protein in *Homo sapiens* were extracted by the AlphaFold database (36) (https://alphafold.ebi.ac.uk/). The tertiary structure of the CCNF protein was further modelled, and the enzyme binding activity and ligand binding site were predicted by PyMOL 2.6 software.

## Results

### Comparison of CCNF gene expression and protein expression levels in pancancer tissues, normal tissues, or adjacent tissues

**Figure 1:**
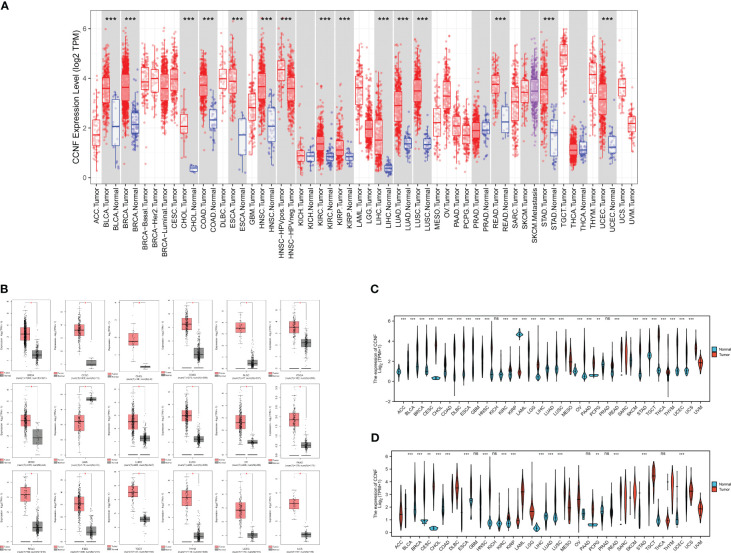
**(A)** Comparison of CCNF expression between pancancer tissues and adjacent normal tissues in the TCGA database (*P<0.05, **P<0.01, ***P<0.001). **(B)** The expression of CCNF was significantly different in cancer tissues from TCGA database and normal tissues from the GTEx database (|Log2FC|>1, *P<0.0001*, log scale: log2 (TPM+1), Jitter Size: 0.4). **(C)** The expression of CCNF in cancer tissues in the TCGA database was compared with that in normal tissues (ns: *P≥0.05*, *P<0.05, **P<0.01, ***P<0.001). **(D)** The expression of CCNF in pancancer tissues and adjacent tissues of TCGA database was compared (ns: *P≥0.05*, *P<0.05, **P<0.01, ***P<0.001).

The expression level of CCNF in 33 kinds of pancancer tissues and adjacent normal tissues was compared by TIMER2.0. The results showed that CCNF was significantly upregulated in cancer tissues *vs*. adjacent normal tissues in 14 types of cancer (*P<0.001*), including bladder urothelial carcinoma (BLCA), breast invasive carcinoma (BRCA), cholangiocarcinoma (CHOL), colon adenocarcinoma (COAD), esophageal carcinoma (ESCA), head and neck squamous cell carcinoma (HNSC), kidney renal clear cell carcinoma (KIRC), kidney renal papillary cell carcinoma (KIRP), liver hepatocellular carcinoma (LIHC), lung adenocarcinoma (LUAD), lung squamous cell carcinoma (LUSC), rectum adenocarcinoma (READ), stomach adenocarcinoma (STAD) and uterine corpus endometrial carcinoma (UCEC) (**Figure 1A**).

The differences in CCNF expression between cancer tissues and normal tissues for 33 kinds of cancer were analyzed by GEPIA2. CCNF was significantly upregulated in BRCA, cervical squamous cell carcinoma and endocervical adenocarcinoma (CESC), CHOL, COAD, lymphoid neoplasm diffuse large B-cell lymphoma (DLBC), ESCA, HNSC, LUAD, LUSC, ovarian serous cystadenocarcinoma (OV), pancreatic adenocarcinoma (PAAD), READ, STAD, testicular germ cell tumors (TGCT), thymoma (THYM), UCEC and uterine carcinosarcoma (UCS) tissues than in normal tissues (*P<0.0001*) (**Figure 1B**).

The differences in CCNF expression between cancer tissues and normal tissues and between cancer tissues and paracancerous tissues for 33 kinds of cancer were analyzed by Xiantao Academy. CCNF was significantly upregulated in adrenocortical carcinoma (ACC), BLCA, BRCA, CESC, CHOL, COAD, DLBC, ESCA, glioblastoma multiforme (GBM), HNSC, KIRC, KIRP, brain lower grade glioma (LGG), LIHC, LUAD, LUSC, OV, PAAD, READ, skin cutaneous melanoma (SKCM), STAD, TGCT, THYM, UCEC and UCS tissues *vs*. normal tissues (*P<0.001*) (**Figure 1C**). CCNF was significantly upregulated in BLCA, BRCA, CHOL, COAD, ESCA, HNSC, KIRC, KIRP, LIHC, LUAD, LUSC, READ, STAD and UCEC tissues *vs*. paracancerous tissues (*P<0.001*) (**Figure 1D**).

In summary, CCNF is significantly upregulated in BRCA, CHOL, COAD, ESCA, HNSC, LUAD, LUSC, READ, STAD and UCEC tissues.

**Figure 2:**
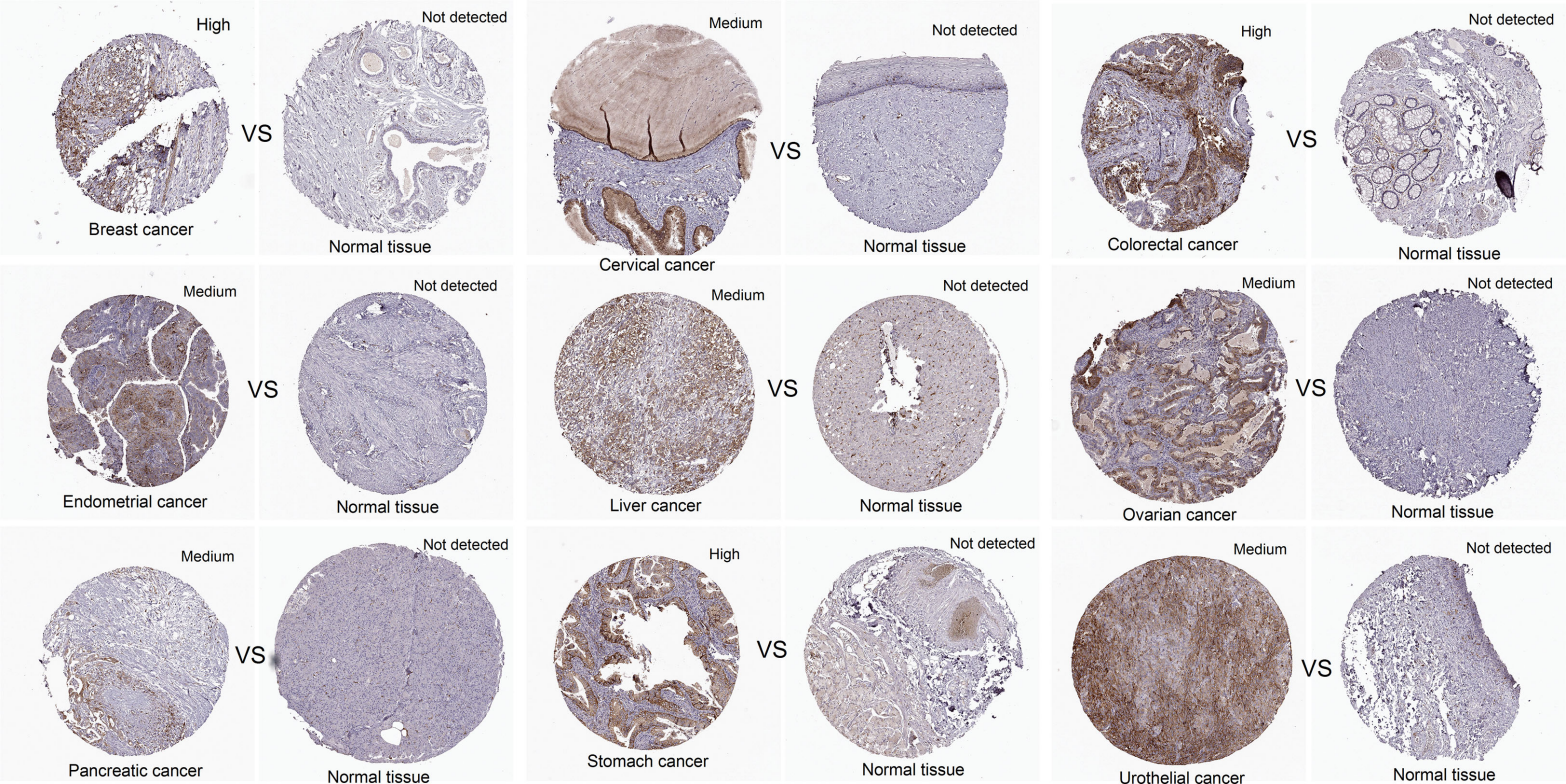
The protein expression levels of CCNF in pancancer tissues and normal tissues were compared via the HPA database. Cancer tissues and normal tissues stained with HPA071600 antibody were selected for immunohistochemistry analysis. Cancer tissues presenting medium or high expression and normal tissues with undetected expression were included.

In addition, the expression level of CCNF protein in cancer tissues and normal tissues for multiple cancer types was compared analysis of immunohistochemical staining data in the HPA database. CCNF protein staining intensity was moderate in cervical cancer, endometrial cancer, liver cancer, ovarian cancer, pancreatic cancer, and urothelial cancer and high in breast cancer, colorectal cancer and stomach cancer. However, CCNF protein staining was not detected in normal tissues (**Figure 2**).

### Correlation of CCNF expression with survival in patients in a pancancer cohort

**Figure 3:**
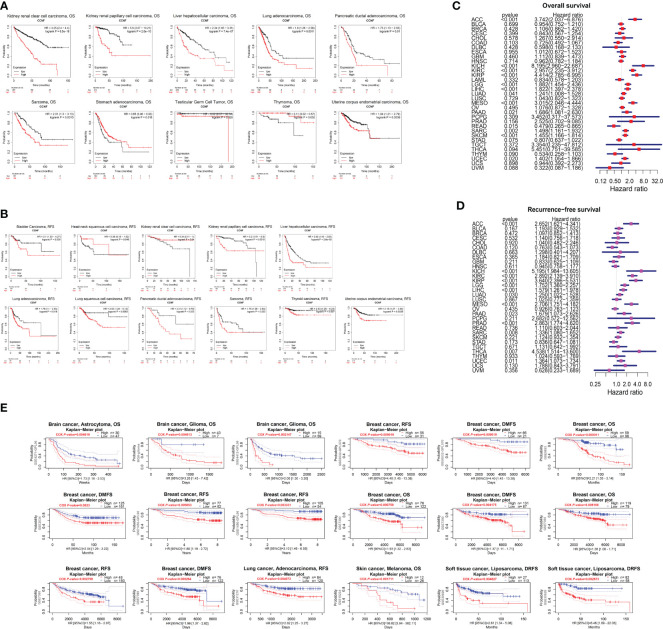
**(A)** Kaplan-Meier-Meier plotter was used to assess the impact of CCNF expression on the OS of patients with 21 types of cancer (*P<0.05*). **(B)** Kaplan-Meier-Meier plotter was used to evaluate the effect of CCNF expression on the RFS of 21 cancer patients (*P<0.05*). **(C)** Cox risk regression analysis of the value of CCNF expression in predicting the OS of cancer patients with 33 cancer types (*P<0.05*, hazard ratio (HR)>1). **(D)** Cox risk regression analysis of the value CCNF expression in predicting the RFS of patients with 33 cancer types (*P<0.05*, HR>1). **(E)** PrognoScan evaluated the effect of CCNF expression on OS, RFS, DMFS and DRFS in pancancer patients (*P<0.01*).

The correlation of CCNF expression with OS and RFS for 21 cancer types was evaluated by Kaplan-Meier Plotter. The overexpression of CCNF was an adverse prognostic factor in terms of OS in patients with KIRC, KIRP, LIHC, LUAD, pancreatic ductal adenocarcinoma (PDAC), Sarcoma (SARC), STAD, TGCT, THYM and UCEC (**Figure 3A**), and an adverse prognostic factor in terms of RFS in patients with bladder carcinoma (BC), HNSC, KIRC, KIRP, LIHC, LUAD, LUSC, PDAC, SARC, thyroid carcinoma (THCA) and UCEC (*P<0.05*) (**Figure 3B**). According to Cox risk regression analysis of data for 33 kinds of cancer, the overexpression of CCNF was an adverse prognostic factor in terms of OS in patients with ACC, kidney chromophobe (KICH), KIRC, KIRP, LGG, LIHC, LUAD, mesothelioma (MESO), PAAD, READ, SARC, SKCM and UCEC (*P<0.05*) (**Figure 3C**), and an adverse prognostic factor in terms of RFS in patients with

ACC, KICH, KIRC, KIRP, LGG, LIHC, LUAD, MESO, PAAD, prostate adenocarcinoma (PRAD), SARC, THCA and UCEC (*P<0.05*) (**Figure 3D**). Therefore, CCNF is often correlated with a poor prognosis in cancer patients.

Furthermore, the effect of CCNF expression on the survival of patients with pancancer was evaluated by PrognoScan. CCNF was an adverse prognostic factor in terms of OS for patients with brain astrocytoma, brain glioma and breast cancer and in terms of RFS for patients with breast cancer and lung adenocarcinoma. In addition, CCNF was an adverse prognostic factor in terms of distant metastasis-free survival (DMFS) in patients with breast cancer and for distant relapse-free survival (DRFS) in patients with soft tissue liposarcoma (*P<0.01*) (**Figure 3E**).

### Effect of sex and racial differences on the expression of CCNF in pancancer

The UALCAN database was applied to compare the expression of CCNF in different sex and race subgroups with in the same cancer cohort. As shown in **Table 1**, the expression level of CCNF in male patients with HNSC (*P=0.0010*) and LUAD (*P=0.0329*) was significantly higher than that in female patients, but CCNF expression in male patients with KIRP (*P=0.0161*) and SARC (*P=0.0064*) was significantly lower than that in female patients with these cancers.

**Table 1:** Effects of sex and race differences on CCNF expression in the same cancer type.

	Patient’s gender		Patient’s race		
	Male	Female	Caucasian	African American	Asian
ACC	2.160 [1.440, 3.115]	2.077 [1.387, 3.555]	2.231 [1.390, 3.711]	NA	NA
BLCA	11.096 [6.915, 15.234]	10.012 [7.591, 13.559]	11.015 [7.030, 14.989]	14.651 [11.802, 17.770] **β**	8.477 [5.535, 11.466] **γδ**
LGG	2.715 [1.958, 3.517]	2.822 [2.025, 3.809]	2.735 [1.990, 3.561]	3.237 [2.105, 4.311]	3.036 [2.767, 3.395]
BRCA	14.854 [11.007, 20.960]	11.104 [7.406, 16.429]	10.437 [7.070, 15.845]	11.542 [7.788, 15.827]	12.590 [9.344, 17.880]
MBC	NA	NA	NA	NA	NA
CESC	NA	NA	1.989 [10.168, 17.492]	12.756 [8.265, 19.296]	14.061 [10.571, 16.199]
CHOL	3.974 [2.541, 4.809]	2.828 [2.285, 4.032]	2.910 [2.433, 4.326]	3.301 [3.052, 3.550]	4.311 [3.265, 4.955]
COAD	10.946 [8.281, 15.304]	12.186 [8.168, 15.781]	11.598 [8.126, 15.879]	11.966 [8.560, 14.942]	11.061 [6.619, 14.510]
EC	13.478 [9.318, 18.591]	13.347 [8.711, 20.808]	12.138 [8.808, 17.383]	22.425 [22.238, 22.462]	14.302 [10.490, 19.306] **γ**
GBM	6.266 [4.127, 9.380]	5.385 [3.586, 8.319]	6.064 [3.857, 8.802]	6.098 [2.366, 9.377]	5.795 [5.398, 6.922]
HNSC	11.727 [8.354, 17.417]	10.535 [7.284, 14.372] **α**	11.456 [7.887, 16.353]	11.960 [9.482, 17.778]	9.354 [6.594, 14.329] **γ**
KICH	0.713 [0.490, 1.039]	0.808 [0.613, 0.988]	0.805 [0.553, 1.407]	0.594 [0.386, 0.784] **β**	1.974 [1.440, 2.508]
KIRC	1.531 [1.201, 2.080]	1.516 [0.967, 1.908]	1.531 [1.136, 2.037]	1.266 [0.969, 1.701]	1.872 [1.338, 2.322]
KIRP	1.086 [0.805, 1.307]	1.418 [0.866, 2.211] **α**	1.146 [0.829, 1.634]	1.037 [0.777, 1.176]	2.733 [1.195, 5.206]
LAML	12.029 [7.987, 16.703]	9.235 [5.411, 13.433]	11.212 [6.763, 15.770]	9.909 [5.793, 13.416]	5.975 [5.644, 6.306]
LIHC	1.521 [0.767, 3.078]	2.230 [1.119, 3.965]	1.595 [0.765, 3.765]	1.642 [1.206, 3.309]	2.070 [1.059, 4.017] **γ**
LUAD	6.754 [3.387, 9.978]	6.152 [3.876, 8.935] **α**	6.244 [3.647, 9.557]	5.733 [3.336, 8.874]	11.444 [7.171, 14.935] **δ**
LUSC	10.037 [7.347, 13.488]	9.809 [6.082, 14.158]	10.206 [7.171, 14.188]	9.710 [7.445, 10.526]	9.172 [8.640, 23.088]
DLBC	14.949 [10.028, 18.396]	13.696 [10.726, 17.277]	14.497 [11.539, 21.021]	NA	14.450 [9.019, 18.319]
MESO	3.471 [2.197, 5.092]	3.645 [1.534, 4.925]	3.542 [1.894, 5.353]	NA	NA
OV	NA	NA	8.758 [6.029, 13.150]	9.034 [6.064, 13.746]	7.096 [5.892, 7.881] **δ**
PAAD	3.088 [2.309, 4.330]	3.260 [2.034, 4.654]	3.129 [2.157, 4.455]	2.652 [2.015, 4.032]	3.780 [3.302, 4.209]
PCPG	2.232 [1.500, 3.027]	2.235 [1.594, 3.312]	2.231 [1.537, 2.994]	2.206 [1.983, 3.383]	2.215 [1.347, 2.766]
PRAD	NA	NA	NA	NA	NA
MPC	NA	NA	NA	NA	NA
READ	12.418 [9.769, 15.480]	11.897 [8.643, 16.306]	10.737 [7.725, 15.585]	12.892 [10.073, 15.358]	NA
SARC	6.335 [3.606, 10.140]	7.742 [4.549, 11.598] **α**	6.842 [3.729, 10.859]	7.727 [6.016, 14.098]	5.721 [3.111, 8.869]
SKCM	10.083 [7.279, 13.636]	9.837 [6.986, 13.754]	10.007 [7.102, 13.636]	NA	13.845 [10.336, 15.287]
STAD	9.916 [6.402, 14.649]	11.411 [7.529, 15.870]	10.238 [5.997, 14.870]	12.572 [9.556, 15.000]	9.631 [6.819, 15.231]
TGCT	NA	NA	29.607 [23.212, 39.737]	30.064 [19.190, 39.444]	39.923 [32.859, 44.088]
THYM	16.191 [9.817, 22.234]	17.015 [8.689, 24.395]	16.718 [10.048, 23.417]	26.352 [22.602, 34.163] **β**	9.022 [5.272, 16.517] **γδ**
THCA	1.151 [0.909, 1.455]	1.125 [0.864, 1.455]	1.163 [0.916, 1.493]	1.032 [0.760, 1.425]	1.163 [0.869, 1.509]
UCS	NA	NA	11.198 [8.490, 14.231]	11.431 [8.476, 14.640]	14.570 [11.129, 15.348]
UCEC	NA	NA	7.588 [4.552, 12.102]	7.768 [4.922, 11.781]	5.634 [3.095, 13.703]
UVM	3.527 [2.671, 4.369]	3.560 [2.536, 4.515]	NA	NA	NA

All data were expressed using interquartile spacing. α: Male vs. Female, P<0.05; β: Caucasian vs. African American, P<0.05; γ: Caucasian vs. Asian, P<0.05; δ: African-American vs. Asian, P<0.05; MBC, Metastatic breast cancer; EC, Oesophageal carcinoma; LAML, Acute myeloid leukemia; PCPG, Pheochromocytoma and paraganglioma; MPC, Metastatic prostate cancer; UVM, Uveal melanoma; NA, Not applicable.

Regarding the expression level of CCNF in BLCA patients, African Americans presented significantly higher expression levels than Caucasians (*P=0.0236*) and Asians (*P=4.450E-05*), and Caucasians presented significantly higher expression levels than Asians (*P=0.0012*). The expression level of CCNF in EC patients was significantly lower in Caucasians than in Asians (*P=0.0487*). The expression level of CCNF in HNSC patients was significantly higher in Caucasians than in Asians (*P=0.0402*). The expression level of CCNF in KICH patients was significantly higher in Caucasian patients than in African American patients (*P<0.05*). The expression level of CCNF in LIHC patients was significantly lower in Caucasians than in Asians (*P=0.0166*). The expression level of CCNF in LUAD patients was significantly lower in African Americans than in Asians (*P=0.0122*). The expression level of CCNF in OV patients was significantly higher in African Americans than in Asians (*P=0.0476*). Regarding the expression level of CCNF in THYM patients, African Americans presented significantly higher expression levels than Caucasians (*P=0.0167*), and Caucasians presented significantly higher expression levels than Asians (*P=0.0327*).

### Correlation between the expression of CCNF and clinicopathological stage

**Figure 4:**
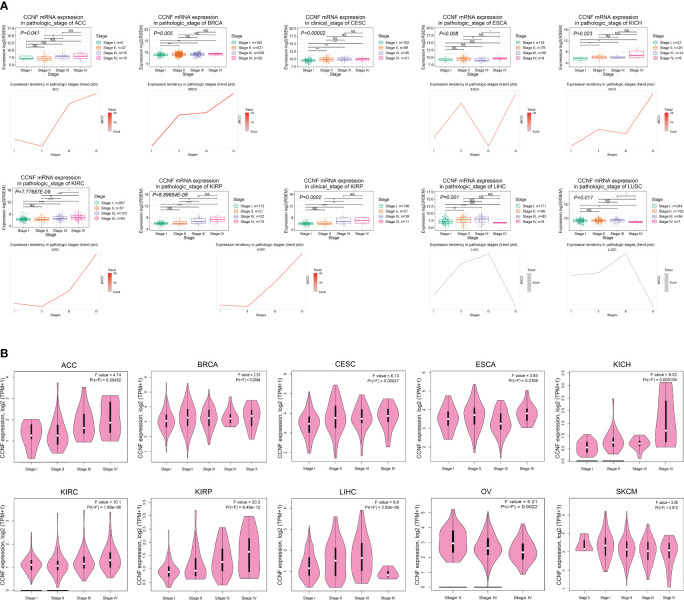
**(A)** Analysis of the correlation between CCNF mRNA expression and the clinical pathological stage for 27 cancer types by GSCA (*P<0.05*). **(B)** The expression of CCNF and the clinical stage of multiple cancers were analyzed by GEPIA2 (*P<0.05*).

The correlation between the mRNA expression of CCNF and the clinicopathological stages of 27 cancer types was analyzed by GSCA. The results indicated that the mRNA expression levels of CCNF in ACC, BRCA, CESC, ESCA, KICH, KIRC, KIRP, LIHC and LUSC were significantly correlated with their clinicopathological stages (*P<0.05*). Specifically, the mRNA expression levels of CCNF in ACC, BRCA, KICH, KIRC and KIRP increased gradually with disease progression, and stage IV tumors presented the highest CCNF expression (**Figure 4A**). The expression of CCNF and the clinical stage of pancancer were analyzed by GEPIA2. The expression of CCNF in ACC, BRCA, CESC, ESCA, KICH, KIRC, KIRP, LIHC, OV and SKCM was significantly different in different clinical stage subgroups (*P<0.05*); the expression of CCNF in ACC, KICH, KIRC and KIRP was the highest in clinical stage IV (**Figure 4B**). In summary, the expression of CCNF in ACC, BRCA, CESC, ESCA, KICH, KIRC, KIRP and LIHC is closely related to the clinicopathological stage. The expression of CCNF in ACC, KICH, KIRC and KIRP was the highest in clinical stage IV tumors, which may contribute to a poor prognosis and cancer progression.

### Effect of CCNF expression on TMB and MSI across cancers

**Figure 5:**
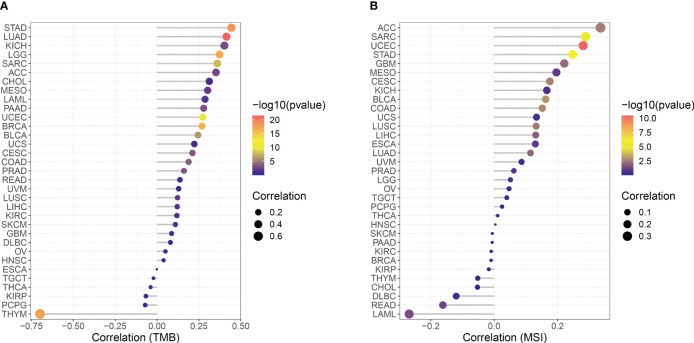
**(A)** Correlation between CCNF expression and TMB in 33 cancer types. **(B)** Correlation between the expression of CCNF and MSI in 33 cancer types. The abscissa represents the correlation value between CCNF expression and TMB and MSI of 33 cancer types, the ordinate represents different cancer types, different colors represent significance levels, and the size of the point represents the size of the correlation coefficient.

The correlation between CCNF expression and TMB and MSI in 33 cancer types was analyzed with R based on the UCSC Xena database. As indicated in **Figure 5A**, there were positive correlations between the expression of CCNF and TMB in ACC (*P=0.0015*), BLCA (*P=5.987E-07*), BRCA (*P=1.786E-17*), CESC (*P=0.0003*), COAD (*P=0.0002*), KICH (*P=0.0009*), KIRC (*P=0.0308*), LAML (*P=0.0228*), LGG (*P=5.771E-18*), LIHC (*P=0.0224*), LUAD (*P=2.857E-22*), LUSC (*P=0.0067*), MESO (*P=0.0068*), PAAD (*P=0.0005*), PRAD (*P=0.0004*), SARC (*P=1.393E-08*), SKCM (*P=0.0181*), STAD (*P=3.602E-19*) and UCEC (*P=2.163E-10*), while there was a negative correlation between the expression of CCNF and TMB in THYM (*P=2.170E-18*). As shown in **Figure 5B**, there were positive correlations between the expression of CCNF and MSI in ACC (*P=0.0025*), BLCA (*P=0.0010*), CESC (*P=0.0022*), COAD (*P=0.0017*), GBM (*P=0.0064*), LIHC (*P=0.0118*), LUAD (*P=0.0097*), LUSC (*P=0.0033*), SARC (*P=3.218E-06*), STAD (*P=1.249E-06*) and UCEC (*P=3.367E-11*), while there were negative correlations between the expression of CCNF and TMB in LAML (*P=0.0271*) and READ (*P=0.0464*).

### Methylation levels of CCNF in pancancer tissues and its effect on patient survival/prognosis and prediction of the microRNA that post transcriptionally regulates CCNF

**Figure 6:**
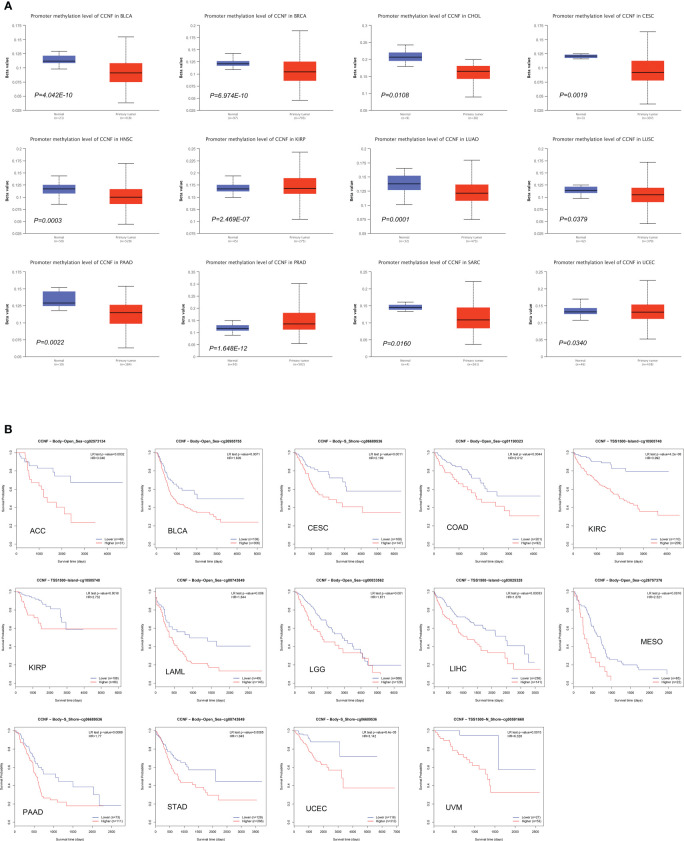
**(A)** Comparison of the methylation level of CCNF between pancancer tissues and normal tissues (*P<0.05*). **(B)** MethSurv was used to analyze the effects of hypermethylation in the CpG site of the CCNF gene on the survival/prognosis of patients in the pancancer cohort (cut-off: HR>1, LR test *P value<0.01*).

The methylation level of CCNF in pancancer tissues was compared with that in normal tissues *via* UALCAN. The methylation level of CCNF in pancancer tissues was significantly lower in BLCA (*P=4.042E-10*), BRCA (*P=6.974E-10*), CHOL (*P=0.0108*), CESC (*P=0.0019*), HNSL (*P=0.0003*), LUAD (*P=0.0001*), LUSC (*P=0.0379*), PAAD (*P=0.0022*), SARC (*P=0.0160*), and UCEC (*P=0.0340*) tissues than in normal tissues but was significantly higher in PRAD (*P=1.648E-12*) and KIRP (*P=2.469E-07*) tissues than in normal tissues (**Figure 6A**).

MethSurv was used to analyze the effects of the methylation levels of CCNF on patient survival/prognosis across cancers. High methylation levels of CCNF resulted in poor survival/prognosis in cancer patients with ACC, BLCA, CESC, COAD, KIRC, KIRP, LAML, LGG, LIHC, MESO, PAAD, STAD, UCEC, and UVM (**Figure 6B**).

The microRNAs that post transcriptionally regulate CCNF based on conservation of the 3’-UTR in *Homo sapiens* were predicted by TargetScanHuman. The aggregate PCT of CCNF and miR-98-5p was 0.94, indicating that miR-98-5p may be involved in the posttranscriptional regulation of CCNF. In addition, the conserved site in the 3’-UTR of miR-98-5p was in the 1786-1793 nt region, and the conserved sequence was GAUGGAG.

### SNV and CNV of the CCNF gene across cancers

**Figure 7:**
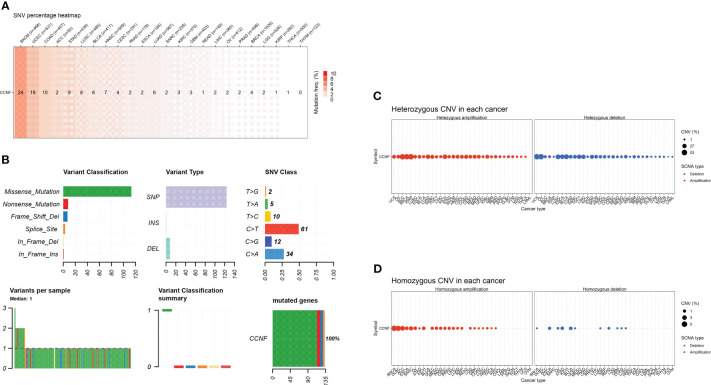
**(A)** The SNV profile of genes of interest in the selected cancers. **(B)** Figure summarizes the SNV classes of the genes of interest in the selected cancers. **(C)** The profiles of heterozygous CNV of the genes of interest in the selected cancers. **(D)** Profile of homozygous CNV of the genes of interest in the selected cancers.

SNV and CNV of CCNF across cancer were analyzed by GSCA. SKCM, UCEC and COAD had the highest numbers of SNVs at 24, 18 and 10, respectively. Missense mutations and single nucleotide polymorphisms (SNPs) were the main variation types, and C>T was the main variation type (**Figures 7A, B**). For CNVs, heterozygous amplification variations of CCNF were most common in KIRP, ACC and BRCA, with amplification percentages of 52.4%, 51.1% and 48.2%, respectively. Loss-of-heterozygosity variations in CCNF were most common in OV, UCS and BLCA, with loss percentages of 53.7%, 53.6% and 40.0%, respectively. Homozygous amplifications of CCNF were most common in BRCA, DLBC and ACC, with amplification percentages of 5.0%, 4.2% and 3.3%, respectively. Homozygous loss of CCNF was most common in BLCA, STAD and ESCA, with loss percentages of 1.5%, 1.4% and 1.1%, respectively (**Figures 7C, D**).

### Correlation between the expression of CCNF and immune cell infiltration, immune score, matrix score, and immune checkpoint gene expression in pancancer

**Figure 8:**
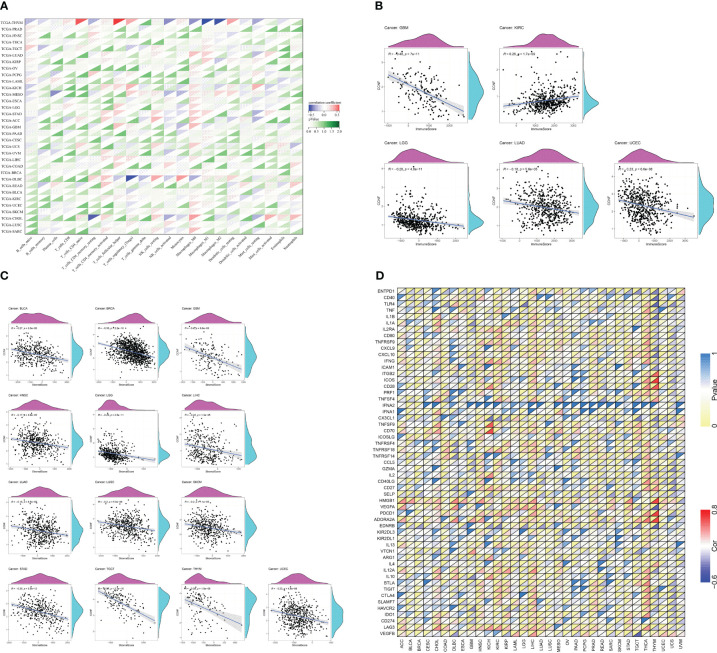
**(A)** Correlation analysis between the expression of CCNF and the infiltration of 22 types of immune cells in 33 cancer types (*P<0.0001*). **(B)** Correlation analysis between the expression of CCNF and immune scores of 33 cancer types (*P<0.0001*). **(C)** Correlation analysis between CCNF expression and the matrix score of 33 cancer types (*P<0.0001*). **(D)** Correlation analysis of CCNF gene and immune checkpoint gene expression in 33 cancer types (*P<0.0001*, |R|>0.5, R: correlation coefficient).

The R language was used to analyze the correlation between CCNF expression and immune cell infiltration. The expression of CCNF in TGCT was positively correlated with the infiltration of naive B cells, and the expression of CCNF in THYM and THCA was positively correlated with the infiltration of plasma cells. The expression of CCNF in BRCA, UCEC, THCA and KIRC was negatively correlated with the infiltration of T cells and resting memory CD4 T cells. The expression of CCNF in STAD, BRCA, LUAD, UCEC and LIHC was positively correlated with the infiltration of T cells and CD4 memory activation. The expression of CCNF in STAD, UCEC, LIHC, THCA, TGCT and SARC was positively correlated with the infiltration of follicular helper T cells. The expression of CCNF in KIRC and BRCA was positively correlated with the infiltration of T-cell regulators (Tregs), while the expression of CCNF in UCEC was negatively correlated with the infiltration of Treg cells. The expression of CCNF in THYM was positively correlated with the infiltration of resting NK cells, while the expression of CCNF in LUSC and COAD was positively correlated with the infiltration of activated NK cells. The expression of CCNF in THYM was negatively correlated with the infiltration of activated NK cells, while the expression of CCNF in BRCA, PAAD, and THCA was negatively correlated with the infiltration of monocytes. The expression of CCNF in BRCA, LUAD, SARC and STAD was positively correlated with the infiltration of M0 macrophages, while the expression of CCNF in THYM was negatively correlated with the infiltration of M0 macrophages. The expression level of CCNF in UCEC, LUAD, THCA, BRCA and STAD was positively correlated with the infiltration of M1 macrophages, while the expression level of CCNF in THYM was negatively correlated with the infiltration of M1 macrophages. The expression level of CCNF in THYM, LIHC and KIRP was negatively correlated with the infiltration of M2 macrophages, while the expression level of CCNF in THYM was positively correlated with the infiltration of resting dendritic cells. The expression of CCNF in HNSC and LUAD was negatively correlated with the infiltration of resting dendritic cells, while the expression of CCNF in KIRC was negatively correlated with the infiltration of activated dendritic cells. The expression of CCNF in LUAD, THYM, KIRC, STAD and BRCA was negatively correlated with the infiltration of resting mast cells, and the expression of CCNF in LUSC was negatively correlated with the infiltration of neutrophils (*P<0.0001*) (**Figure 8A**).

The R language was used to analyze the expression of CCNF and the immune score and matrix score across cancers. The expression of CCNF was negatively correlated with the infiltration of immune cells in GBM, LGG, LUAD and UCEC (*P<0.0001*), while the expression of CCNF was positively correlated with the infiltration of immune cells in KIRC (*P<0.0001*) (**Figure 8B**). The expression level of CCNF was negatively correlated with the infiltration of stromal cells in BLCA, BRCA, GBM, HNSC, LGG, LIHC, LUAD, LUSC, SKCM, STAD, TGCT, THYM, and UCEC (*P<0.0001*) (**Figure 8C**).

The R language was used to analyse the correlation of the CCNF gene and immune checkpoint gene across cancers. There was a significant negative correlation between the CCNF gene and the EDNRB gene (R=-0.5751, *P=7.861E-12*), VEGFA gene (R=-0.6083, *P=2.161E-13*), TLR4 gene (R=-0.5862, *P=2.484E-12*), and CD40 gene (R=-0.5405, *P=2.221E-10*) in THYM. There was a significant positive correlation between the CCNF gene and the ADORA2A gene (R=0.7431, *P=3.788E-22*), PDCD1 gene (R=0.7560, *P=2.855E-23*), HMGB1 gene (R=0.7394, *P=7.757E-22*), CD28 gene (R=0.6638, *P=1.915E-16*), ICOS gene (R=0.7617, *P=8.523E-24*), and ITGB2 gene (R=0.5928, *P=1.224E-12*) in THYM. The CCNF gene was positively correlated with the ADORA2A gene (R=0.5337, *P=6.792E-39*) in THCA. The CCNF gene was positively correlated with the CD70 gene (R=0.7168, *P=1.874E-11*) and TNFSF9 gene (R=0.7168, *P=5.970E-09*) in KICH. The CCNF gene was significantly negatively correlated with the CX3CL1 gene (R=-0.5162, *P=1.078E-05*) in KICH (**Figure 8D**).

### PPI network and GO and KEGG enrichment analyses of CCNF and related genes

**Figure 9:**
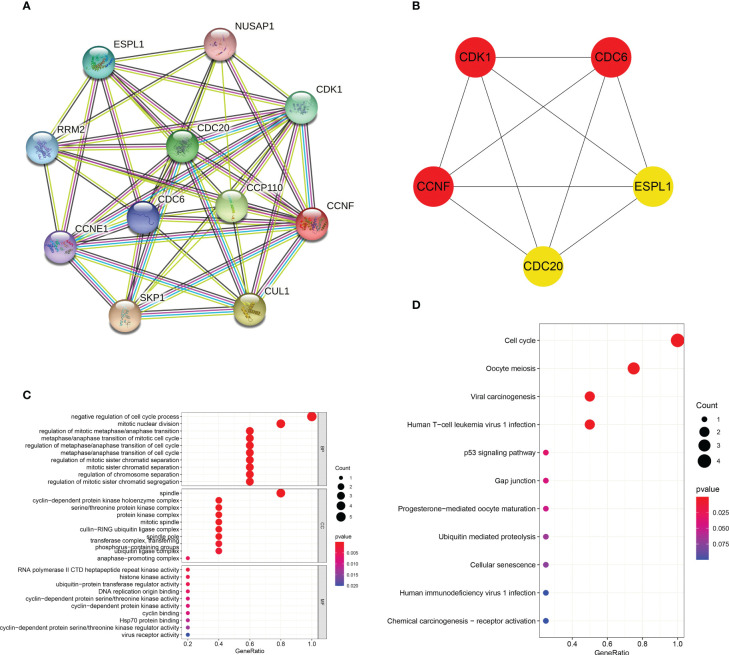
**(A)** Analysis of the PPI interaction network of CCNF through STRING and Cytoscape. **(B)** The top 5 hub genes predicted through the cytoHubba app for the PPI interaction network of CCNF. **(C)** GO enrichment analysis of the top 5 hub genes was conducted *via the* R language. **(D)** The KEGG signaling pathways enriched in the top 5 hub genes were analyzed with the R language.

The PPI interaction network of CCNF was analyzed by STRING and Cytoscape. The PPI interaction network was composed of 11 coding genes (CCNF, CDC6, CDC20, CDK1, NUSAP1, CCP110, CUL1, SKP1, CCNE1, RRM2, and ESPL1) and 46 edges (**Figure 9A**). The top 5 hub genes were predicted by the cytoHubba plugin, and CCNF, CDK1 and CDC6 were most closely related (**Figure 9B**). GO enrichment analysis consists of three categories: biological process (BP), cellular component (CC) and molecular function (MF). Negative regulation of the cell cycle process was the main enriched BP of the top 5 hub genes, and spindle was the main enriched CC term. The main enriched MF term was RNA polymerase II CTD heptapeptide repeat kinase activity (*P<0.05*) (**Figure 9C**). KEGG signaling pathway analysis results reflect the network of interactions, reactions and relationships between molecules. There were 7 KEGG signaling pathways enriched in the top 5 hub genes, and the cell cycle signaling pathway was the main enriched pathway (*P<0.05*) (**Figure 9D**).

### Prediction of basic physical and chemical properties and secondary structure of CCNF protein

**Figure 10:**
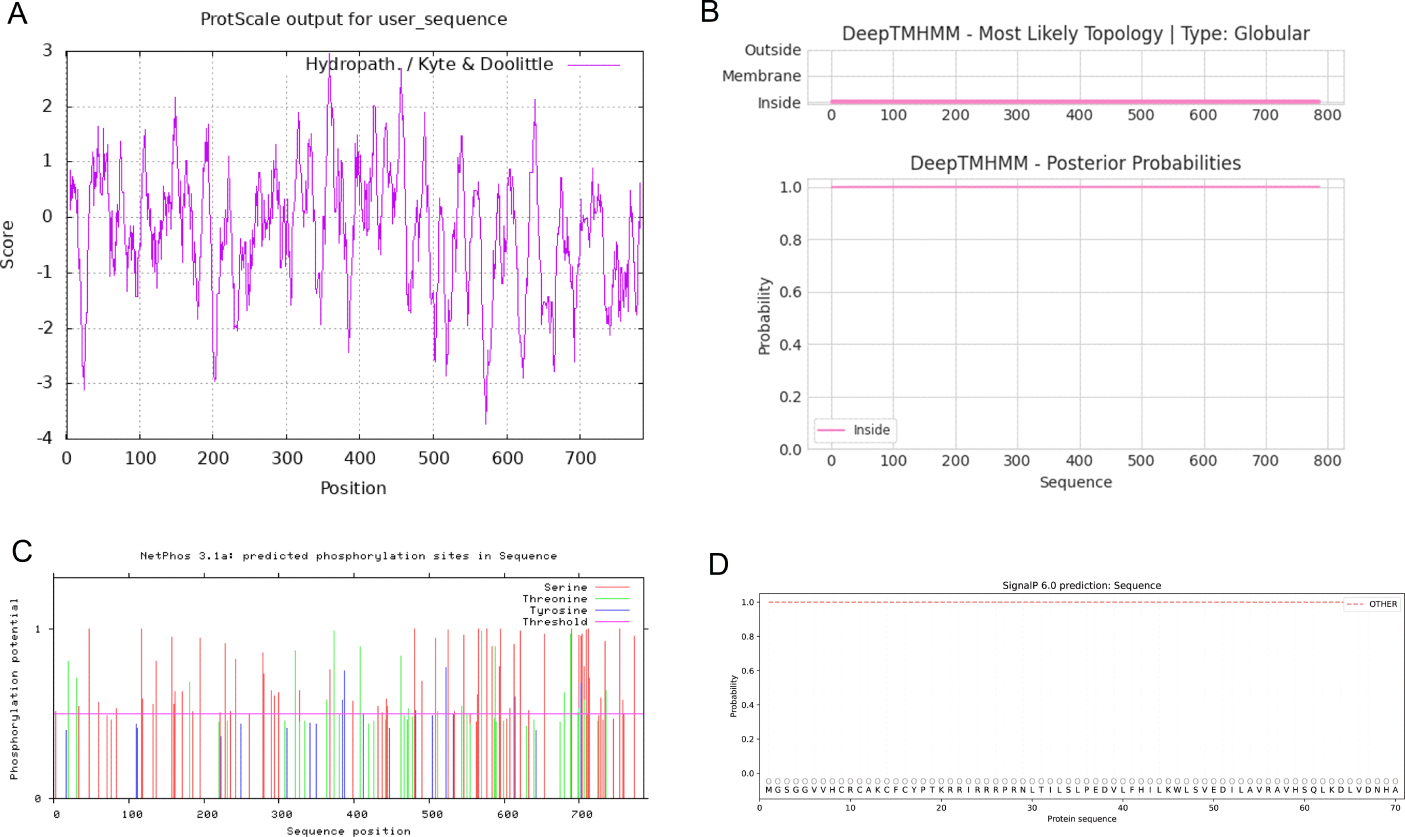
**(A)** The hydrophobicity of the CCNF protein. The abscissa is the sequence position, and the ordinate is the scale value of the amino acid. (Hphob./kyte & Doolittle: positive value indicates hydrophobicity, negative value indicates hydrophilicity). **(B)** The transmembrane topology of the CCNF protein. **(C)** The phosphorylation sites of serine, threonine and tyrosine residues of the CCNF protein. **(D)** Signal peptide of CCNF protein.

According to the prediction of the basic physical and chemical properties of the CCNF protein, the total number of amino acids in the CCNF protein was 786, the proportion of isoleucine was the highest, the content was 12.3%, the total molecular weight was 87639.81, the theoretical PI value was 5.92, the total number of positively charged residues (Arg+Lys) was 84, the total number of negatively charged residues (Asp+Glu) was 98, the molecular formula was C_3854_H_6112_N_1082_O_1174_S_39_, and the total number of atoms was 12261. At a wavelength of 280 nm, all paired cysteine residues are assumed to form cystine with an extinction coefficient of 1.099, and all cysteine residues are assumed to decrease with an extinction coefficient of 1.076. Instability index: 51.10, which is unstable protein. Aliphatic index: 88.33, Grand average of hydropathy (GRAVY): -0.245, maximum hydrophilic position: 358, score: 2.956, maximum hydrophobic position: 572, score: -3.722, was hydrophilic protein (**Figure 10A**). The transmembrane topology of the CCNF protein has no transmembrane region (**Figure 10B**). There were 62 phosphorylation sites for serine residues, 20 phosphorylation sites for threonine residues, and 6 phosphorylation sites for tyrosine residues (**Figure 10C**). The signal peptide of the CCNF protein is not a signal peptide (**Figure 10D**). The CCNF protein included 361 α-helices (45.93%), 54 β-folds (6.87%), 31 β-turns (3.94%) and 340 random coils (43.26%) according to the predicted secondary structure of CCNF protein coils.

### Prediction of the tertiary structure, enzyme binding activity, and ligand binding sites of the CCNF protein

**Figure 11:**
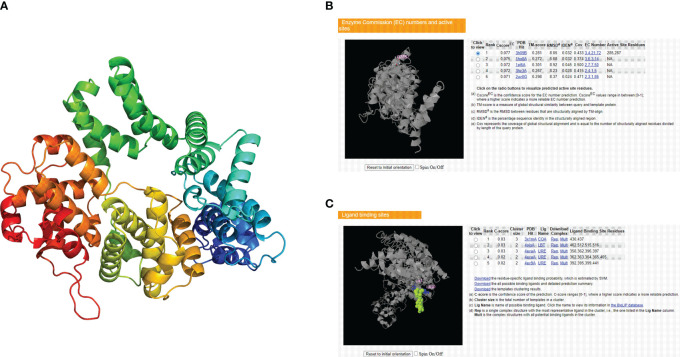
**(A)** AlphaFold database and PyMOL software predictions of the tertiary structure of the CCNF protein. **(B)** PyMOL software was used to predict the enzyme binding sites of the CCNF protein. **(C)** PyMOL software was used to predict the ligand binding sites of the CCNF protein.

The tertiary structure data for the CCNF protein were extracted by the AlphaFold database and visualized by PyMOL software (**Figure 11A**). The enzyme binding activity and ligand binding site of the CCNF protein were predicted by PyMOL software. The best model was selected according to the C-score. The higher the value of the C-score is, the higher the reliability of the model. The following parameters were extracted: C-score of enzyme binding activity of CCNF protein: 0.077, active site: 285, 287, C-score of ligand binding: 0.03, and binding site: 430, 437 (**Figures 11B, C**).

## Discussion

In recent years, pancancer analysis has become very important in revealing commonalities among cancer types and facilitating individualized treatment. It has been applied not only in the discovery of new tumor markers and the development of new anticancer drugs (37) but also in the online analysis of the underlying molecular mechanism and clinical prognostic value of a gene (38). For the first time, we analyzed the differential expression of CCNF across cancers and its correlation with clinical prognosis. In our study, CCNF was generally highly expressed in 33 cancer types, and the results were cross-validated with the TIMER 2.0, GEPIA2 and Xiantao Academic online databases. The expression levels of CCNF in BRCA, CHOL, COAD, ESCA, HNSC, LUAD, LUSC, READ, STAD and UCEC were significantly higher than those in adjacent tissues or normal tissues, suggesting that overexpression of CCNF is an adverse prognostic factor for cancer. At the same time, our study also found that the expression level of CCNF protein was high in many cancer tissues, especially in breast cancer, colorectal cancer, and stomach cancer tissues. CCNF, as a key component of the ubiquitin proteasome, may maintain genome integrity by mediating the degradation of intracellular proteins, while overexpression of CCNF causes dysregulation of DNA replication, repair and cell cycle checkpoints, thereby inducing the progression of cancer cells (6, 39). However, CHANG S C et al. (40) reported that CCNF reduced the activity of cancer cells and inhibited the migration and invasion of BRCA cancer cells by downregulating the expression of the RRM2 gene. Although their conclusions are contrary to ours, CCNF and its encoded proteins may be potential biomarkers for the diagnosis of cancer.

In the era of precision medicine for cancer, pancancer biomarkers and clinicopathological features can be used to assess the condition and prognosis of cancer patients more comprehensively than traditional markers alone (41). Our results show that CCNF is an adverse prognostic factor in terms of OS and RFS in patients with KIRC, KIRP, LIHC, LUAD, SARC and UCEC, which was also confirmed in the Cox hazard regression analysis. The correlation between the expression level of CCNF and the clinicopathological stage of multiple cancer types was analyzed *via* GSCA and GEPIA2 online, and the results also showed that the overexpression of CCNF was associated with advanced disease and a poor prognosis, suggesting that CCNF could be used as a potential pancancer biomarker. Increasing evidence has shown that the instability of cancer genomes differs between males and females, which leads to differences in mutation rates during tumor evolution in these groups (42). DONG M et al. (43) and WANG Y et al. (44) showed that when patients are younger than 65 years old, the morbidity and survival rates of male patients are lower than those of female patients in a variety of cancers, and male sex is an independent risk factor for cancer development and distant metastasis. This conclusion is consistent with our findings. The expression level of CCNF in male patients is generally higher than that in female patients, which also suggests that CCNF is a potential pancancer therapeutic target influenced by sex. There are similar differences in patients grouped by race (45, 46). In our study, the expression level of CCNF in Caucasians with BLCA and THYM cancer was significantly lower than that in African Americans but significantly higher than that in Asians. Therefore, we will consider the influence of sex and race more carefully in future cancer treatment.

Immune checkpoint inhibitors (ICIs) have facilitated great achievements in the field of cancer immunotherapy, and TMB and MSI are the only two factors that have been approved by the Food and Drug Administration (FDA) as indicators of the response to ICI treatment (47). TMB reflects the number of gene mutation sites in tumor cells, while MSI is caused by a defect in mismatch repair (MMR) function. Tumors with high TMB and MSI are more readily infiltrated with immune cells, which facilitates a stronger antitumor immune response (48, 49). In our study, the CCNF expression level was significantly positively correlated with TMB and MSI status in ACC, BLCA, CESC, COAD, LIHC, LUAD, LUSC, SARC, STAD, and UCEC and was closely related to the expression levels of many immune checkpoint genes in THYM and KICH. These results suggest that CCNF and its encoded proteins may be biomarkers for predicting the effect of ICI treatment in cancer patients. DNA methylation can control the expression of certain genes without changing gene sequences, which is an important form of genome epigenetic modification (50). This is mainly reflected by the observation that the hypermethylation of CpG islands in the promoter region leads to the silencing of tumor suppressor genes, while the hypomethylation of CpG sites in the gene body promotes the activation of oncogenes. However, this methylation level can be changed (51). Studies have shown that abnormal DNA methylation is an important cause of genomic instability in cancer cells, and methylation levels constantly affect the occurrence and progression of tumors (52, 53), consistent with our results. The hypomethylation of CCNF in BLCA, BRCA, CHOL, CESC, HNSC, LUAD, LUSC, PAAD, SARC and UCEC leads to the overexpression of the CCNF gene. In addition, we have shown that high methylation levels of CCNF in ACC, COAD, KIRC, KIRP, LAML, LGG, LIHC, MESO, STAD, and UVM patients lead to poor survival/prognosis. Therefore, the methylation level of CCNF is also a very important factor affecting the survival/prognosis of patients with different cancer types, suggesting that combined assessment of methylation level and expression level of CCNF may become a new method for the diagnosis and treatment of cancers in the future.

The tumor microenvironment (TME) is a complex entity composed of cancer cells, stromal cells and immune cells, and the invasion of cancer cells is the main factor affecting the occurrence and development of tumors and immune escape (54, 55). In recent years, the antitumor effect of infiltrating immune cells on the TME and the response to treatment have been widely studied (56), and comprehensive antitumor therapy, including treatment with immunotherapies that enhance the infiltration of innate and adaptive immune cells in the TME, is very important (57). Our results showed that THYM cancer was mainly infiltrated by resting NK cells, plasma cells and resting dendritic cells. Activated memory CD4 T cells, follicular helper T cells, M0 macrophages and M1 macrophages infiltrate widely in many cancer types, which is consistent with the conclusion of ZUO S et al. (55). Although the high heterogeneity of the TME affects the ability of immune cells to infiltrate, the extent of T-cell and macrophage infiltration is an important prognostic factor for many cancer types. In addition, CCNF overexpression also reduced the infiltration of immune cells and stromal cells in GBM, LGG, LUAD and UCEC cancers, thereby promoting the growth of tumor tissues. We predicted the microRNA that targets the CCNF gene, and the results showed that miR-98-5p may be involved in the posttranscriptional modification of the CCNF gene. According to previous studies, miR-98-5p not only inhibits the growth and invasion ability of gastric cancer cells (58) and thyroid papillary cancer cells (59) but also enhances the sensitivity to cisplatin and paclitaxel. Moreover, the downregulation of miR-98-5p often predicts the later clinical stage and distant metastasis of non-small cell lung cancer (NSCLC) (60), indicating that miR-98-5p may be a microRNA that negatively regulates the CCNF gene. PPI interaction network analysis showed that the CCNF gene, CDK1 gene and CDC6 gene are closely related, indicating that these genes, as key regulators of the G2/M or G1/S checkpoint of the cell cycle, promote the occurrence and progression of many cancers. CCNF is a potential pancancer biomarker and immunotherapeutic target (61, 62).

## Conclusion

The CCNF gene is overexpressed in BRCA, CHOL, COAD, ESCA, HNSC, LUAD, LUSC, READ, STAD and UCEC cancers and is also an adverse prognostic factor in terms of OS and RFS in many cancers, suggesting that it promotes the progression of many cancers and indicates a poor prognosis. In addition, the hypomethylation of the CCNF gene promotes its own expression, leading to decreased infiltration of immune cells and stromal cells, and the expression level of CCNF clearly differs between patients grouped by sex and race. miR-98-5p negatively regulates the expression of the CCNF gene at the posttranscriptional level. Therefore, CCNF and its encoded proteins are potential biomarkers and immunotherapeutic targets for pancancer.

## Data availability statement

The original contributions presented in the study are included in the article/supplementary material. Further inquiries can be directed to the corresponding authors.

## Ethics statement

Ethical review and approval was not required for the study on human participants in accordance with the local legislation and institutional requirements. Written informed consent for participation was not required for this study in accordance with the national legislation and the institutional requirements. Ethical review and approval was not required for the animal study because no ethical approval or informed consent was required in this study due to the public availability of data in the TCGA database.

## Author contributions

Conceptualization: RW; methodology: LW, HL, MX, and CZ; software: LW and MX; validation: LW, HL, and JL; formal analysis: LW, CZ, and SW; investigation: LW and HL; data curation: RW and SW; writing-original draft preparation: LW, HL, and MX; writing-review and editing: RW; visualization: RW and SW; supervision: RW; project administration: RW. All authors contributed to the article and approved the submitted version.
